# Chinese cross-cultural adaptation and validation of the Well-being Numerical Rating Scales

**DOI:** 10.3389/fpsyt.2023.1208001

**Published:** 2023-10-05

**Authors:** Qing Luo, Chunqin Liu, Ying Zhou, Xiaofang Zou, Liqin Song, Zihan Wang, Xue Feng, Wenying Tan, Jiani Chen, Graeme D. Smith, Francesca Chiesi

**Affiliations:** ^1^School of Nursing, Guangzhou Medical University, Guangzhou, China; ^2^The Third Affiliated Hospital of Guangzhou Medical University, Guangzhou, China; ^3^School of Health Sciences, Caritas Institute of Higher Education, Hong Kong SAR, China; ^4^Department of Neuroscience, Psychology, Drug, and Child’s Health (NEUROFARBA), University of Florence, Florence, Italy

**Keywords:** well-being, translation, psychometric evaluation, reliability, validity, item response theory, classical test theory

## Abstract

**Introduction:**

Well-being is a multi-domain concept that involves measuring physical, psychological, social, and spiritual domains. However, there are currently few multi-domain and comprehensive well-being instruments available. In addition, measures that do exist customarily contain a vast number of items that may lead to boredom or fatigue in participants. The Well-being Numerical Rating Scales (WB-NRSs) offer a concise, multi-domain well-being scale. This study aimed to perform the translation, adaptation, and validation of the Chinese version of WB-NRSs (WBNRSs-CV).

**Methods:**

A total of 639 clinical participants and 542 community participants completed the WB-NRSs-CV, the Single-item Self-report Subjective Well-being Scale (SISRSWBS), the World Health Organization Five-item Well-Being Index (WHO-5), the 10-item Perceived Stress Scale (PSS-10), and the Kessler Psychological Distress Scale (K10).

**Results:**

High internal consistency and test-retest reliability were obtained for both samples. Additionally, WB-NRSs-CV was positively associated with SISRSWBS and WHO-5 and negatively associated with PSS-10 and K10. In the item response theory analysis, the model fit was adequate with the discrimination parameters ranging from 2.73 to 3.56. The diffculty parameters ranged from −3.40 to 1.71 and were evenly spaced along the trait, attesting to the appropriateness of the response categories. The invariance tests demonstrated that there was no difference in WB-NRSs-CV across groups by gender or age.

**Discussion:**

The WB-NRSs-CV was translated appropriately and cross-culturally adapted in China. It can be used as a rapid and relevant instrument to assess well-being in both clinical and non-clinical settings, with its utility for well-being measurement and management among the Chinese people.

## 1. Introduction

Well-being can be connected to many areas of human life and is also one of the most important indicators reflecting mental health (1). Moreover, research studies consistently indicate that a higher level of well-being is associated with positive health outcomes, such as reduced risk of ill health, enhanced quality of life, and longer survival rates (2–4). It has been acknowledged that, with its potential to prevent disease and promote overall health status and quality of life (QoL), well-being is receiving increasing interest worldwide (5). Despite its growing popularity, there exists limited consensus within the scientific community on the definition of well-being. For instance, well-being has initially been characterized as a pleasant emotion, while others have argued that it refers specifically to autonomy, positive relationships, engagements, accomplishments, and the pursuit of a purposeful life (6). These inconsistent findings have raised the interest of scholars in enhancing their knowledge and understanding the nature and measurements of well-being.

Indeed, considerable effort has been made to enhance the understanding of the nature of well-being in recent decades, leading to the emergence of numerous theoretical methods and conceptualizations that have been specifically developed to properly define and measure positive health and well-being (7). Four theories have been highlighted across the varying conceptualizations of wellbeing including hedonic well-being, eudaimonic well-being, wellness, and QoL (8). Among these theories, hedonic well-being provides the most proverbial subjective well-being model, consisting of life satisfaction, positive affect, and negative affect (9). Eudaimonic well-being, on the other hand, places significant emphasis on personal true potential, including personal growth, a sense of autonomy, positive relations with others, and purpose in life (10). The wellness approach is firmly rooted in focusing on an individual’s optimal functioning, encompassing physical, psychological, social, and spiritual aspects, with an emphasis on the idea that well-being is more than the absence of illness (11). Finally, QoL is closely related to both wellness and well-being, and the term QoL is often used interchangeably with wellness or well-being within the academic literature (12). The attention these four approaches have received across a variety of academic disciplines might explain the emergence of multiple conceptualizations of well-being; that is, hedonic and eudaimonic well-being have emerged primarily from psychology and sociology, QoL from medicine, and wellness from counseling (8). Thus, well-being can be viewed as a broad and multi-domain construct, and ideally, measures of well-being should incorporate multi-domain items associated with a range of aspects of well-being conceptualizations or theoretical models.

To date, several well-being measurement instruments, such as the Satisfaction with Life Scale (SWLS) (9), the Positive and Negative Affect Schedule (PANAS) (13), the World Health Organization Well-Being Index (WHO-5) (14), and the Social Well-Being Scale (SWBS) (15), have been developed synchronously based on the four categories of conceptualizations mentioned. Those tools have been fully validated across different populations around the world and are widely utilized in clinical and general settings (16), yet each has different limitations. First, most well-being measurement scales solely focus on one or a few aspects of well-being conceptualizations or theoretical models, thereby limiting the ability to fully measure the multi-domain constructs of well-being. For example, the SWBS only contains one domain (social) and focuses on the eudaimonia facet of well-being, while the WHO-5, SWLS, and PANAS specifically target the hedonic facets of well-being (17). However, employing multiple well-being instruments may add variability to research results, making it more difficult to synthesize findings. As such, it has been suggested that it might be more meaningful to examine well-being as a parsimonious and comprehensive overarching construct rather than attempting to reduce it into component parts (18). Researchers have been continuously calling for a new generation of well-being scales to be developed from a multi-domain perspective. Second, to our knowledge, although several measurements, such as the Mental Physical Spiritual Well-Being Scale (MPS, 30 items) (19), the Bio-psycho-social-spiritual Inventory (BIOPSSI, 41 items) (20), and the WHO Quality of Life Assessment (WHOQOL-100) (21), evaluate well-being across multiple domains, these instruments customarily consist of a vast number of items that are more likely to make participants experience boredom, loss of interest, or fatigue, particularly for older adults and patients. To overcome these limitations and capture more comprehensive information on well-being, a practical and brief instrument that captures multiple domains of well-being would be more useful and favorable in clinical and research settings.

Recently, a novel and brief well-being measurement tool, the Well-being Numerical Rating Scales (WB-NRSs), was developed and validated in Italian and Canadian populations through the item response theory (IRT) approach (22). The WB-NRSs were developed based on the most recent definition of health put forward by WHO: “Health is not only the absence of disease or disability but also the state of complete physical, psychological, social, and spiritual well-being” (23, 24). Accordingly, based on the multidimensional definition of health, four paramount components of well-being were identified, and general well-being was considered simultaneously to provide an all-encompassing representation of individual well-being (22). Hence, the WB-NRSs provide a comprehensive assessment of well-being, including physical, mental, social, spiritual, and general well-being. In addition, the WB-NRSs comprise five numerical rating scales that not only offer the advantages of ease of use and visualization, short administration time, high comprehensibility, and simplicity of scoring, but they are also amenable to large, multivariate scale surveys (25). Although the WB-NRSs demonstrate good discrimination ability and appropriate response categories in each item, it remains a relatively new scale and requires further validation within different social and cultural settings. To the best of our knowledge, no scale has been developed to encompass all the domains of well-being in Mainland China. Therefore, the purpose of our study was to culturally adapt and validate the WB-NRSs in the Chinese population.

Classical test theory (CTT) and item response theory (IRT) are two major methodologies to test the psychometric properties of instruments. Classical test theory is a well-established paradigm that is widely employed by researchers to develop and validate instruments. However, technological developments have enabled the use of IRT analysis, offering more stringent psychometric methods and potential advantages over CTT (26). The Item response theory adopts a mathematical approach to derive scores based on a Logit model and focuses on the relationship between personal ability and level on the construct measured by the scale and their probability of responding positively to each item, whereas CTT is based on the relationship between individual location on the construct and their observed total scores on the scales (27). Accordingly, CTT cannot offer an absolute representation of the psychometric properties; it primarily provides information about how responses to different items are correlated, which comes with certain limitations (28, 29). Furthermore, only a single standard error value can be obtained for a whole group using CTT, whereas IRT provides a unique standard error estimation for each participant (30). In contrast, IRT can bring items with high discriminatory abilities, generate rich item information on the factor structure, and provide valuable information about the difficulty and discrimination ability of each item. Therefore, it provides a powerful tool to develop, evaluate, and refine a new generation of health outcomes instruments (31, 32). Taken together, this study aimed to evaluate the cross-cultural psychometric properties of the WB-NRSs among the Chinese population using an approach based on both CTT and IRT. We anticipated that the findings of this study would provide a sound and rapid assessment tool for the measurement and management of well-being in the Chinese population, which is potentially useful for the researchers interested in assessing well-being within this specific demographic.

## 2. Method

In the adaptation and validation processes, this study followed the International Test Commission’s (ITC) Guidelines for Translating and Adapting Tests (second edition) (33).

### 2.1. Adaptation process

After having obtained consent from the research group that developed the WB-NRSs, the translation and cross-cultural adaptation process was carried out based on the principles of Brislin’s model of forward and backward translation (34). Initially, two bilingual translators, who were knowledgeable about research into well-being and fluent in both the Chinese and English languages, independently translated the questionnaires into Chinese and generated two translated versions of the WB-NRSs (WB-NRSs-1 and WB-NRSs-2). Subsequently, the third translator (YZ), proficient in both Chinese and English, compared the two translated versions with the original scale to check whether there were any ambiguities and discrepancies in expression, sentences, and meanings. If any discrepancies between the two translations were identified, the third translator would discuss these differences with the two translators to reach a consensus via video conference. If necessary, adjudication was performed by an expert committee. This expert committee comprised of five experts with relevant doctorates and rich and varied research experience across psychology, sociology, and instrument development. Through a consensus approach, this committee discussed inappropriate expressions and reconciled the translations. For instance, in the wording of item 4 (spiritual well-being), there was a discrepancy in expressions between the two Chinese versions. In this item, “spiritual well-being” was translated with the word “精神幸福感” by one translator; however, another translator translated it as “灵性幸福感.” In this instance, the third translator discussed these differences with the two translators in an attempt to synthesize the results. However, the disagreements could not be reconciled. As such, the expert committee was involved in adjudication. The experts considered that translating “spiritual well-being” with the Chinese word “灵性幸福感” was inaccurate because, in Chinese, “灵性幸福感” can be easily understood as “religious well-being” and a transcendent experience of religion in the Chinese cultural context. Therefore, the word “灵性幸福感” was substituted by “精神幸福感,” and finally, the two versions were merged into a harmonized Chinese version (WB-NRSs-3). Subsequently, the Chinese version of WB-NRSs-3 was back-translated into English by another two bilingual translators with English linguistic backgrounds who had not seen the original English version, resulting in two independent back-translation versions (WB-NRSs-4 and WB-NRSs-5). A conceptual, semantic, and content equivalence assessment of the two back-translated versions (WB-NRSs-4 and WB-NRSs-5) and the original version was performed by the expert committee and the five translators mentioned above. Furthermore, both the draft and back-translated versions of the questionnaire were sent to the original authors to ensure that they were sufficiently close to the original version. Since the original WB-NRSs required minimal language translation, no further rounds of translation were deemed necessary. Finally, a preliminary version of the WB-NRSs-CV was established.

Before the formal survey, the WB-NRSs-CV was pilot-tested with 30 patients with chronic diseases and 30 community residents to evaluate the expressions, instructions, and response format of the scales for clarity. A dichotomous scale with the words “clear” and “unclear” was used to assess the participants’ understanding of the scale, and the time taken to complete the questionnaire was recorded. The result showed that the WB-NRSs-CV was in line with the Chinese language expression habits, clearly understood, and took approximately 3 min to complete.

### 2.2. Psychometric test of the WB-NRSs-CV

#### 2.2.1. Participants and procedures

This was a cross-sectional study. From December 2021 to June 2022, participants were recruited through convenience sampling from the clinical and non-clinical settings in Guangzhou, Guangdong province, to ensure sampling across the full range of well-being, which was better for examining the psychometric properties of WB-NRSs-CV. Face-to-face and paper-and-pencil interviewing (PAPI) with structured questionnaires were conducted to collect the data. The clinical sample was recruited from the chronic wards of two tertiary hospitals. While patients were hospitalized, the healthcare providers or investigators extended an invitation to those who met the eligibility criteria. The inclusion criteria included: (1) aged 18–90 years, (2) being diagnosed with at least one of the 10 most common chronic diseases based on the International Classification of Diseases, 10th Revision (ICD10), (3) hospitalization for over 3 days, (4) and being able to communicate verbally and willing to participate. The non-clinical participants were community residents recruited from five districts in Guangzhou. Before the commencement of the study, posters were exhibited on residents’ bulletin boards, advertisements were published on the community homepage, and WeChat invitations were carried out by the community workers to draw the interest of prospective participants. The criteria for inclusion were as follows: (1) age 18–90 years, (2) having lived in the community for at least 1 year, and (3) able to communicate verbally and willing to participate. Their common exclusion criteria included individuals diagnosed with neuropsychiatric diseases or severe cognitive impairment, combined with severe heart, hepatic, renal, pulmonary, or brain dysfunction, and those who withdrew from this study. Participants from both samples were grouped into sets of 10–30 individuals per group, with each group managed by 2–4 investigators. Structured questionnaires were handed out to participants and collected immediately by the investigators. The investigators explained the purpose of the investigation, ensured that participants understood the questions and response options, and provided any necessary assistance to participants who may have difficulty reading or understanding the questions. A completeness check was conducted after the questionnaire was provided by the investigators. Finally, each participant received a gift as a token of appreciation. A total of 1,208 respondents were recruited; 27 (18 community residents and 9 patients) were excluded due to extreme values and incomplete responses. As such, 1,181 respondents (542 residents and 639 patients) were available for the final analysis. Among the 542 residents, the average age was 44.60 (16.18) years, with a range of 21–87 years, and 76.4% were women. For the 639 patients, the average age was 62.10 (14.85) years, with a range of 19–90 years, and 50.2% were women.

#### 2.2.2. Data collection

##### 2.2.2.1. Demographics

The demographic characteristics of the participants include age, sex, marital status, education level, and income, as well as clinical variables such as types of chronic disease, time since the first confirmed diagnosis, and daily medicine intake.

##### 2.2.2.2. The Chinese version of the Well-being Numerical Rating Scales

The WB-NRSs-CV consists of five items on a 10-point numerical rating scale, ranging from 1 (absolute distress) to 10 (complete well-being). Each item on the scale measures a specific domain: physical, mental, social, spiritual, and general well-being. A higher score relates to a higher level of well-being (22).

##### 2.2.2.3. The single-item self-report subjective well-being scale

The SISRSWBS scale has been extensively used in empirical well-being studies (35). It only consists of one item, namely, “At present, how satisfied are you with your life?.” A score between 1(strongly disagree) and 7 (strongly agree) is assigned to the item, directly assessing individuals’ happiness levels. The higher the score recorded, the higher the well-being level.

##### 2.2.2.4. World Health Organization well-being index

WHO-5 is a generic global scale that was designed to assess an individual’s level of well-being in the previous 2 weeks and has been translated into over 30 languages and validated in many countries (36). The scoring for each item ranges from 0 (at no time) to 5 (all the time). The five items’ scores were summed to create a total score (range: 0–25), with higher scores indicating a higher level of well-being. The psychometric properties of the WHO-5 were also examined in the Chinese population, and favorable results were documented (Cronbach’s α > 0.81 in multiple samples) (37).

##### 2.2.2.5. Perceived stress scale-10 item

The perceived stress scale-10 item (PSS-10) is a 10-item scale designed to screen for the degree of subjective stress (38). Respondents assessed how often they experienced the respective feelings of stress within the last month with a 5-point rating scale (0 = never, 4 = very often). Total scores range from 0 to 40, with scores≥14 suggesting moderate stress levels. The Chinese version of PSS-10 has excellent internal consistency (Cronbach’s α = 0.86) and test–retest reliability (Cronbach’s α = 0.68), with convergent associations with other measures of stress (39).

##### 2.2.2.6. The Kessler psychological distress scale

The K10 is a 10-item measure designed as a 5-point rating scale (1 = none of the time, 5 = all of the time), assessing individual psychological distress levels in the last 4 weeks (40). The total scores were obtained by summing the response to each item (range: 10–50), with higher values representing a greater level of psychological distress. The Chinese version of the K10 showed good reliability and validity in the Chinese population (41).

### 2.3. Statistical analysis

All statistical analyses were performed with SPSS 26.0 and R software, and the significance level was set to 0.05 for all statistical tests. Descriptive statistics were used to analyze the demographic and clinical characteristics of the respondents. Psychometric property analyses of the WB-NRSs-CV followed four steps: (1) reliability and validity analysis derived from CTT; (2) explore factor analysis (EFA) and confirmatory factor analysis (CFA); (3) IRT analysis; and (4)multi-group confirmatory factor analysis (MG-CFA).

In the current study, we examined the reliability and validity of the patients’ and community residents’ samples. The reliability of the WB-NRSs-CV was tested using Cronbach’s alpha(α) coefficient and test–retest reliability. An α value of 0.7 or higher is deemed acceptable. Studies have suggested that a test-retest period of 4 weeks or 30 days is appropriate (42, 43). In this study, the Pearson correlation coefficient (R) was employed to assess the test–retest correlation during the follow-up period of 4 weeks, and significance was attributed to *R* values exceeding 0.75 and *p*-values lower than 0.05 (44). The validity was analyzed by criterion-related validity, which was investigated by comparing it against a related instrument, the SISRSWBS, WHO-5, PSS-10, and K10.

When we executed the EFA, CFA, and IRT analyses, we merged the two samples (542 residents and 639 patients). Before executing the IRT analysis, it is necessary to confirm whether the five items of the WB-NRSs-CV violate unidimensionality and local independence assumptions. We used the full sample to perform both EFA and CFA in our study to confirm unidimensionality. Local independence was defined as item scores that do not correlate when holding the latent trait constant and evaluated by examining Yen’s Q3 statistic (45). High Yen’s Q3 statistics (greater than 0.36) were flagged in the current study, indicating a high risk of systematic fitting problems and being considered as possible local dependence (LD) (46). As such, an EFA, including the scree plot criteria (47) and Horn’s parallel analysis (48), was performed to determine the number of extracted factors. Differences in the magnitude of the first eigenvalue and second eigenvalue (ratio at least 4:1), scree plots, and factor loadings were considered good indicators of the unidimensional assumption (32). Next, a single-factor CFA model was estimated, and the model fit was evaluated based on the following indices: the Root Mean Square Error of Approximation (RMSEA), Standardized Root Mean Square Residual (SRMR), Comparative Fit Index (CFI), and Tucker-Lewis Index (TLI), with values of the RMSEA and SRMR under 0.05 and values of the TLI and CFI above 0.95 reflecting adequate and good fit (49).

Then, on the basis of unidimensionality and local independence, IRT analysis was performed to examine whether any of the items of the WB-NRSs-CV with the intended ordering of response category thresholds and the ability of items to discriminate among individuals with different well-being levels. Choosing the appropriate model and evaluating its fit are indispensable prerequisites to confirming that the model is fit for the data. Considering the WB-NRSs-CV’s response format (Likert-type scale), we can select from several IRT models that have been developed for ordered polytomous response items (26): Samejima’s Graded Response Model (GRM), Generalized Partial Credit Model (GPCM), and Rating Scale Model (RSM). Log-likelihood (LL), Akaike Information Criterion (AIC), and Bayesian Information Criterion (BIC) were used to compare and choose the optimal model, with lower values indicating improved fit (50). Similarly, the IRT model fit was assessed using various indices, namely, marginal likelihood information statistics (M_2_) and the associated RMSEA. The limit value for RMSEA is 0.05, and a value less than this indicates goodness of fit (51). Then, marginal maximum likelihood estimation was used to obtain the item parameters of the best model, including the discrimination (a) and difficulty (b) parameters. Taking the GRM as an example, each item has a parameter, indicating the ability of an item to discriminate among individuals across levels of the latent trait (i.e., well-being, denoted as theta). The value of a corresponds to different levels of discrimination: low discrimination = 0–0.64; moderate discrimination = 0.65–1.34; high discrimination = 1.35–1.69; very high discrimination ≥1.70 (52). Besides, the amount of information was also used to provide another measure of the discriminatory power and precision of an item, which was represented graphically by the item information function (IIF). Thus, a higher value of the a parameter and a higher amount of item information indicate a greater ability of the item to distinguish between respondents with different levels of well-being (28). The b parameters indicate the level of the latent trait where there is a 0.5 probability that a participant will endorse a specific item (51), and the number of b parameters for each item is one fewer than its number of response options. If the b parameters are evenly spaced along a wide range of traits, the item categories will provide better differentiation and variability in measuring well-being. In addition, the item characteristic curve (ICC) was used to provide visual information regarding the item characteristics. The ICC is a logistic function that models the relationship between an individual’s response to an item and his level on a certain scale and expresses how the probability of selecting an item changes as a function of the item’s a and b parameters (53).

Finally, we performed an MG-CFA across study participants from various groups (i.e., different groups of gender and age) to test the measurement invariance of these groups. This testing included the equality of the overall factor structure (configural invariance), the equality of item factor loadings (metric invariance), and the equality of item intercepts (scalar invariance) (54). A gender comparison was carried out between male and female participants, and three groups were established for age: younger adults (18 ≤ Age ≤ 44), middle-aged people (45 ≤ Age ≤ 59), and older people (Age ≥ 60).

## 3. Results

### 3.1. Descriptive statistics

Descriptive and item analyses (including range, means, and standard deviations) were conducted for each item of the WB-NRSs-CV. The normality of each item of the WB-NRSs-CV was examined as a preliminary step prior to analysis. All the skewness and kurtosis indices ranged between −1 and 1, suggesting that there were no departures from a normal distribution (Table 1).

**Table 1:** Descriptions, item-total correlations (with item deleted), and factor loadings of the Chinese version of the Well-being Numerical Rating Scales (WB-NRSs-CV).

Variable	Range	Mean	SD	Skewness	Kurtosis	Item-total correlation	Factor loading
WB-NRSs-CV	5–49	33.47	8.85	−0.57	−0.28	/	/
Physical WB	1–10	6.52	2.00	−0.42	−0.20	0.79	0.87
Psychological WB	1–10	6.84	2.07	−0.58	−0.17	0.82	0.89
Social WB	1–10	7.32	1.98	−0.671	0.00	0.79	0.87
Spiritual WB	1–10	6.55	1.97	−0.34	−0.34	0.80	0.88
General WB	1–10	6.23	2.08	−0.04	−0.76	0.82	0.89

### 3.2. Classical test theory analysis

#### 3.2.1. Reliability

High internal consistency of the WB-NRSs-CV was observed in both the patient group (Cronbach’s α = 0.921) and the community resident group (Cronbach’s α = 0.939). Test-retest reliability for both groups within a time interval of 4 weeks was also adequate, at 0.878 for patients and 0.885 for residents, respectively.

#### 3.2.2. Validity

Criterion-related correlation validity analysis revealed that the WB-NRSs-CV was significantly and positively associated with SISRSWBS and WHO-5 (*r* = 0.884, 0.846, *p* < 0.01; *r* = 0.772, 0.820, *p* < 0.01), and negatively associated with PSS-10 and K10 (*r* = −0.819, −0.823, *p* < 0.01; *r* = −0.592, −0.613, *p* < 0.01) in both the patient and the community samples, respectively (Table 2), indicating both good internal consistency and effectiveness.

**Table 2:** Bi-variate correlation between the WB-NRSs–CV and the other variables in the study.

	1	2	3	4	5	6	7	8	9	10
1. Physical WB	/	0.775^**^	0.786^**^	0.706^**^	0.777^**^	0.900^**^	0.734^**^	0.707^**^	−0.444^**^	−0.512^**^
2. Psychological WB	0.705^**^	/	0.802^**^	0.722^**^	0.750^**^	0.901^**^	0.764^**^	0.696^**^	−0.0553^**^	−0.552^**^
3. Social WB	0.670^**^	0.669^**^	/	0.759^**^	0.791^**^	0.923^**^	0.749^**^	0.697^**^	−0.598^**^	−0.584^**^
4. Spiritual WB	0.681^**^	0.729^**^	0.696^**^	/	0.703^**^	0.864^**^	0.694^**^	0.647^**^	−0.473^**^	−0.517^**^
5. General WB	0.702^**^	0.733^**^	0.683^**^	0.740^**^	/	0.900^**^	0.738^**^	0.718^**^	−0.580^**^	−0.582^**^
6. WB-NRSs-CV	0.859^**^	0.884^**^	0.850^**^	0.882^**^	0.885^**^	/	0.820^**^	0.772^**^	−0.592^**^	−0.613^**^
7. WHO-5	0.703^**^	0.814^**^	0.657^**^	0.753^**^	0.752^**^	0.846^**^	/	0.735^**^	−0.659^**^	−0.638^**^
8. SISRSWBS	0.715^**^	0.834^**^	0.697^**^	0.798^**^	0.805^**^	0.884^**^	0.864^**^	/	−0.575^**^	−0.612^**^
9. PSS-10	−0.643^**^	−0.805^**^	−0.668^**^	−0.731^**^	−0.717^**^	−0.819^**^	−0.829^**^	−0.838^**^	/	0.807^**^
10. K10	−0.668^**^	−0.814^**^	−0.649^**^	−0.723^**^	−0.726^**^	−0.823^**^	−0.843^**^	−0.854^**^	0.916^**^	/

Below Diagonal = patients sample (*N* = 639); above Diagonal = community sample (*N* = 542). WB-NRSs-CV = The Chinese Version of the Well-being Numerical Rating Scales; WHO-5 = World Health Organization Well-being Index; SISRSWBS = Single-item Self-report Subjective Well-being Scale; PSS-10 = Perceived Stress Scale; K10 = Kessler Psychological Distress Scale.

**p < 0.01.

### 3.3. Item response theory analysis

#### 3.3.1. Unidimensionality

The scree plot of eigenvalues in the EFA strongly suggested a one-factor structure, with the first factor accounting for 77% of the total variance. The ratio of eigenvalues of the first and second factors was 11.2, which was much higher than the required minimum of 4. Item-total correlation values ranged from 0.79 to 0.82, and all factor loadings were greater than 0.83 (Table 1). The one-factor structure presented a very satisfactory fit to the data: χ^2^*/*df = 3.397, CFI = 0.997, TLI = 0.994, RMSEA = 0.045 [95% CI, 0.023–0.069], SRMSR = 0.14. Based on the results from EFA and CFA, it was evident that the WB-NRSs-CV met the assumption of unidimensionality.

#### 3.3.2. Local independence

Based on the one-factor structure, the local independence of each item pair was explored (Table 3). All Q3 statistics were below 0.36, indicating the absence of covariation between items and a low risk of systematic model fit issues. Overall, these results showed that, after accounting for the dominant factor, the items on the scale hardly suffered from LD.

**Table 3:** The Q3 statistics of the WB-NRSs-CV.

Item	1	2	3	4	5
1. Physical WB	1				
2. Psychological WB	−0.207	1			
3. Social WB	−0.243	−0.182	1		
4. Spiritual WB	−0.276	−0.221	−0.139	1	
5. General WB	−0.150	−0.290	−0.259	−0.287	1

#### 3.3.3. Model choice and model fit

The GRM showed a better fit to the data compared to the GPCM and GRSM. Therefore, we selected the GRM as the optimal model (Table 4). Additionally, the fit for the GRM was deemed adequate (*M*_2_ = 17.002, df = 5, *p* < 0.010; RMSEA = 0.045; SRMSR = 0.014; TLI = 0.996; CFI = 0.998).

**Table 4:** The model fit indices.

Model	−2LL	AIC	BIC
GRM	−10007.28	20114.56	20368.26
GPCM	−10056.25	20212.50	20466.21
RSM	−10151.04	20330.08	20401.12

GRM, Graded Response Model; GPCM, Generalized Partial Credit Model; RSM, Rating Scale Model; −2LL, −2Log-Likelihood; AIC, Akaike’s Information Criterion; BIC, Bayesian Information Criterion.

#### 3.3.4. Item parameter evaluation

**Figure 1:**
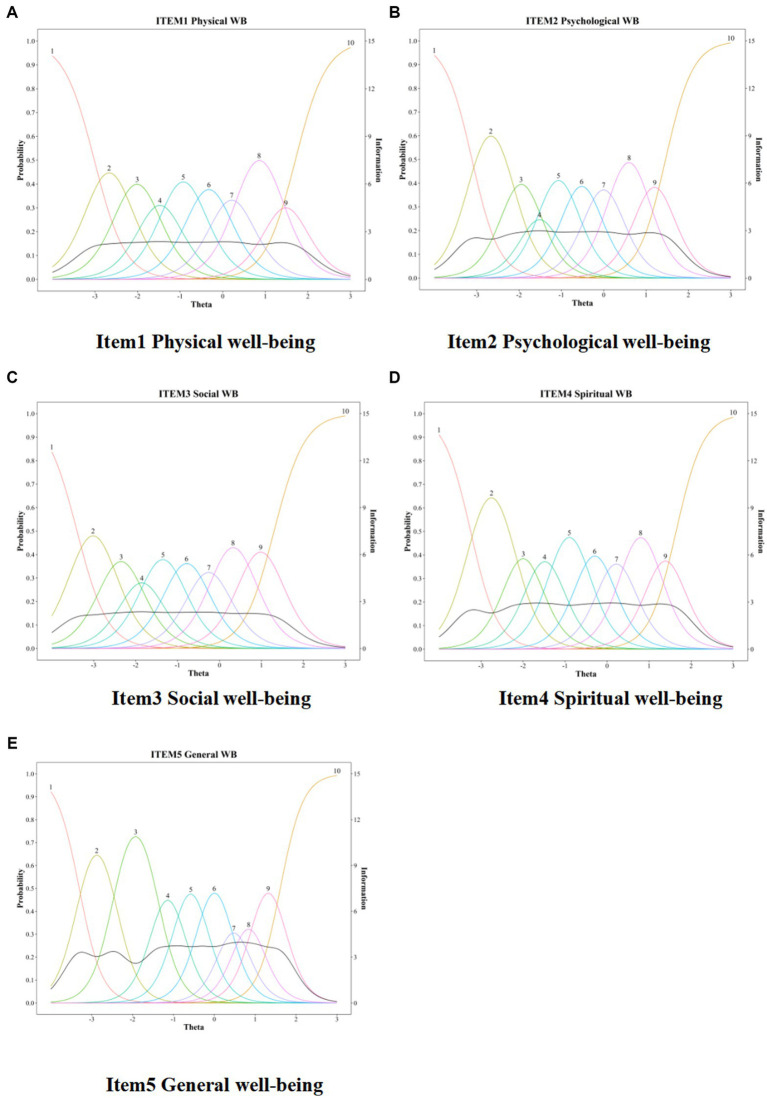
Item characteristic curves (ICCs) and item information functions (IIFs) for five items of WB-NRSS-CV. The x axis “Theta” represented the range of latent trait of well-being, the left y axis “Probability” indicated the probability of endorsing a response option (multicolored line), and the right y axis “Information” represented the amount of information (black line) yielded by the item at each trait level. **(A)** Item 1 physial well-being. **(B)** Item 2 psychological well-being. (C) Item 3 social well-being. **(D)** Item 4 spiritual well-being. **(E)** Item 5 general well-being.

All parameter estimates from the GRM calibration are presented in Table 5. Each item had a very high value of discrimination parameter (a), ranging between 2.73 and 3.56, with items 2, 4, and 5 showing the highest values. Hence, all items could distinguish well between low and high well-being levels of respondents in physical, mental, social, spiritual, and general well-being domains. Particularly, psychological, spiritual, and general well-being items were the best-performing ones. The five items of WB-NRSs-CV demonstrated difficulty parameters (b) that span the level of well-being from −3.40 to 1.71. The b1 and b2 were roughly from about 3.00 to 2.00 SDs below the mean theta (fixed to *M* = 0.00, SD = 1.00, by default), while the b3, b4, and b5 were roughly from about 2.00 to 0.30 SDs below the mean. Additionally, b6 and b7 were roughly around the mean, and b8 and b9 were from 0.60 to 2.00 SDs above the mean (Table 5). These b parameters were evenly spaced, implying that WB-NRSs-CV was robust in discriminating a person with well-being below or above the mean theta, specifically for those participants at lower levels of well-being. However, it should be noted that the b parameter span should ideally cover from 3 SDs below to 3 SDs above the mean. Consequently, it can be observed that the five items failed to adequately cover the highest levels of the trait and had some limitations. Similar findings could also be obtained from the ICCs and IFFs shown in Figure 1. The item curves of the five items were steep and centralized within the latent trait range of −3.00 to 2.00. Moreover, each response category threshold for all items followed the expected ordering and showed a specific level of trait for which the probability of choosing it was higher. For instance, respondents with low well-being (theta around −3.00) had a higher probability of selecting option 1 or 2, and high well-being respondents (theta around 1.00) had a higher probability of endorsing option 8 or 9. These findings indicated that all items behaved appropriately, although the highest trait was insufficiently covered by the b parameters. The IIFs demonstrated that items 1–5 conveyed a large amount of information from −3.00 to 1.50 SDs, but a dramatic plunging in the range from about 1.50 SDs to 3.00 SDs above the mean appeared and provided nearly little or no information for latent trait values inside of that range (Figure 1). As such, all the items provided the most reliable information when participants reported relatively low levels of latent well-being, while the WB-NRSs provided less reliable information when they reported relatively higher levels of well-being.

**Table 5:** Discrimination and difficulty parameters of the WB-NRSs-CV.

	Discrimination	Difficulty								
Item	a	b1	b2	b3	b4	b5	b6	b7	b8	b9
Physical WB	2.76	−3.02	−2.32	−1.71	−1.24	−0.61	−0.04	0.46	1.26	1.71
Psychological WB	3.09	−3.11	−2.22	−1.68	−1.35	−0.78	−0.26	0.25	0.94	1.46
Social WB	2.73	−3.40	−2.63	−2.06	−1.64	−1.06	−0.50	−0.01	0.66	1.30
Spiritual WB	3.09	−3.25	−2.26	−1.74	−1.23	−0.57	−0.02	0.47	1.13	1.64
General WB	3.56	−3.31	−2.44	−1.41	−0.87	−0.29	0.30	0.65	1.03	1.61

a = Discrimination Parameter; b_i_ = Difficulty Parameters.

### 3.4. Invariance test

Table 6 showed that the configural model (M1) exhibited a reasonably good fit to the data across gender and age groups, suggesting an equivalent factor structure across these groups. This model provided a baseline to compare subsequent models. We then sequentially tested the metric invariance model (M2) and the scalar invariance model (M3), one after the other. The results demonstrated that the changes in fit indicated by these models did not substantially decline (i.e., ΔCFI ≤ 0.010 and ΔRMSEA ≤ 0.015) (55). This suggests that the WB-NRSs-CV has the same meaning and function across both male and female participants, as well as across different age groups, including younger adults, middle-aged individuals, and older people. Thus, it may be efficiently applied to Chinese subjects of different genders and age groups for comparable scores.

**Table 6:** Test of measurement invariance across gender and age.

Model	χ^2^	df	CFI	RMSEA[90%CI]	△CFI	△RMSEA
Gender						
M1 (configural)	27.148	10	0.996	0.054 [0.030, 0.079]		
M2 (metric)	38.190	14	0.994	0.054 [0.034, 0.075]	0.002	0.00
M3 (scalar)	53.252	18	0.987	0.065 [0.041,0.089]	0.007	−0.011
Age						
M1 (configural)	37.578	15	0.995	0.062 [0.037,0.087]		
M2 (metric)	59.527	23	0.992	0.064 [0.044, 0.084]	0.003	−0.002
M3 (scalar)	91.025	31	0.984	0.077 [0.051,0.099]	0.008	−0.013

CFI, Comparative Fit Index; RMSEA, Root Mean Square Error of Approximation.

## 4. Discussion

This study aimed to validate the cross-cultural psychometric properties of the WB-NRSs-CV in the Chinese population. This research is novel in that it combines CTT and IRT psychometric methods to comprehensively assess a scale focusing on the well-being of a large sample composed of clinical and community participants. The findings demonstrated that the WB-NRSs-CV is a reliable and valid instrument for precisely and efficiently assessing well-being across clinical and non-clinical settings. Furthermore, the investigation established that all items in the WB-NRSs-CV exhibited equivalent functionality across different genders and age groups.

Utilizing the CTT method, we verified the psychometric properties of WB-NRSs-CV within both patient and community samples, respectively. The outcomes demonstrated a significant positive correlation between the WB-NRSs-CV and SISRSWBS, as well as WHO-5, and a negative association with PSS-10 and K10 in both groups. The higher the individual’s perceived stress and psychological distress, the lower their sense of well-being. Our findings correspond with previous research studies that have pointed out a link between higher well-being and lower levels of perceived stress and psychological stress (56–58). It is possible that an individual may view stress or psychological distress as debilitating, negatively affecting their well-being. Moreover, the WB-NRSs-CV showed high internal consistency and re-test reliability, with Cronbach’s alpha varying from 0.878 to 0.939 (re-test to internal consistency), suggesting the scale had good stability and validity over time. These findings derived from CTT analysis have not been reported in the study conducted by Bonacchi et al. (22). Additionally, our EFA and CFA analyses yielded results in line with the one-factor structure of the WB-NRSs proposed by the original authors (22), which represents a good cultural adaptation of this scale.

In this study, the calibration analyses suggested that the items of the WB-NRSs-CV had a satisfactory fit in the IRT model. Each item demonstrated remarkably high discriminatory power (a > 2.73). All b parameters for the items effectively encompassed the range of well-being from −3.40 to 1.71. Additionally, these parameters were uniformly distributed along the trait, indicating the suitability of the response categories. Altogether, the WB-NRSs-CV is robust in discriminating individuals based on various aspects and levels of well-being, specifically for those participants with lower levels of well-being. It is worth noting that the original scale performed well as an ideal for measuring well-being in patients with cancer and liver disease with cirrhosis and non-clinical individuals, but it was unable to adequately cover the highest levels of the trait (b parameters between −3.62 and 2.41) (22), which were similar to the results obtained in our study. We advocate that the observed effectiveness of the WB-NRSs within a limited range of the latent trait does not undermine the internal validity of its score interpretations. As previously argued, one superior factor of IRT is the ability to estimate reliability at any point along the latent trait continuum, in contrast to the overall reliability index typically seen in CTT. Ideally, the difficulty parameters would be distributed from −3 to +3, but in the IRT-based psychometric literature, it is not uncommon to observe such insufficient coverage of a latent trait due to practical application problems (59, 60). Besides, it might also be attributed in part to cultural variations among the study participants. Influenced by the Chinese Confucian culture, the respondents may display a tendency to avoid selecting the highest scoring answers (i.e., 9 or 10 points). Thus, it may be difficult to obtain data from people with extremely high levels of well-being. More research is therefore required to verify the difficulty range of WB-NRSs; such research should be conducted with larger, more inclusive samples and within different cultural settings, which may contribute to the construction of far-reaching scales (61).

In addition, an exploration of potential cross-cultural variations could be an avenue for future research. This is crucial to enable researchers to confidently compare outcomes across diverse cultural samples, ensuring that the WB-NRSs retain a consistent construct and functionality. Future studies are needed to use the structural method of invariance to allow for a more direct comparison between the English, Italian, and Chinese versions of the WB-NRSs. Moreover, it is possible that discrepancies exist between patients and residents in terms of environmental factors (e.g., patients resided in the hospital setting while residents were community-based), psychological, and physical aspects, which might contribute to the different response distributions in the WB-NRSs scale between the groups to differentiate the two groups. This represents a potential direction for future research as well.

Nevertheless, we believe that our study has clinical and research implications. To date, there has been no culturally appropriate, comprehensive, and concise instrument to assess well-being among the Chinese people. The WB-NRSs-CV is an empirically tested scale that has a potential role to play in measuring well-being levels accurately and providing further support for the development of tailored and targeted interventions for the Chinese people. Due to its brevity, the WB-NRSs-CV facilitates minimizing the burden on respondents and takes less administration time in comparison with other similar scales. For these reasons, it could be usefully adopted to assess well-being in communities and clinical practice. Moreover, with the availability of different validated versions of the WB-NRSs, research into cross-cultural comparisons could be undertaken in the future.

## 5. Limitations

Notwithstanding the reported strength of psychometric properties, there remain some limitations within the current study. First, bias cannot be completely avoided due to the use of convenience sampling. Second, the current sample only comprises patients with chronic diseases and community residents, which may result in bias in population selection, and as a result, the findings may not be generalized to other populations, such as other patients with other diseases. Further research with more diverse populations could be included to explore the scale’s applicability. Over and above, considering the terms “psychological well-being” and “spiritual well-being” are easily understood as similar concepts in the Chinese cultural context, it is possible that adding more detailed and comprehensible instruction could help participants understand and discriminate the specific meaning of each item. Finally, given that most individuals prefer to report themselves as having a good level of well-being, there is a potential problem of social desirability bias in responses when using the scale. It could be considered to use this self-report scale in combination with other assessment methods, such as observations by families, partners, psychological therapists, or nurses.

## 6. Conclusion

Overall, the WB-NRSs-CV was translated appropriately and cross-culturally adapted for use in the Chinese population. The scale could be used as a comprehensive, concise, and reliable well-being instrument to rate physical, psychological, spiritual, social, and general well-being. One attraction of the WB-NRSs-CV is its brevity, potentially causing less burden for respondents. More broadly, it represents a promising instrument for future research into assessing well-being among the Chinese population in clinical settings and communities.

## Data availability statement

The raw data supporting the conclusions of this article will be made available by the authors, without undue reservation.

## Ethics statement

The studies involving humans were approved by the Ethics Committee of Guangzhou Medical University (No. 202201008). The studies were conducted in accordance with the local legislation and institutional requirements. The participants provided their written informed consent to participate in this study.

## Author contributions

QL and CL have contributed to this article by coordinating data collection, elaborating the theoretical framework, and writing the manuscript. YZ and XZ have contributed to this article through the coordination of data collection, the writing of the manuscript, and supervision. XZ, LS, ZW, WT, XF, and JC have contributed to this article through data collection. GS has contributed to this article through a critical revision of the manuscript. FC has contributed to the adaptation process. All authors contributed to the study’s conception and design. All authors contributed to the article and approved the submitted version.
