# Supplementary material for: Chinese cross-cultural adaptation and validation of the Well-being Numerical Rating Scales
Source: Front Psychiatry. 2023 Oct 5;14:1208001. doi: 10.3389/fpsyt.2023.1208001 (PMC10585061; doi:10.3389/fpsyt.2023.1208001)
Supplement: Supplementary file 1 [file Data_Sheet_1.PDF]

Supplementary Material

Chinese Cross-Cultural Adaptation and Validation of the Well-being Numerical Rating Scales（WB-NRSs）

Qing Luo[1†], Chunqin Liu[1†], Ying Zhou[1*], Xiaofang Zou[2], Liqin Song[1], Zihan Wang[1], Xue Feng[1], Wenying Tan[1], Jiani Chen[1], Graeme D. Smith[3]

[1]School of Nursing, Guangzhou Medical University, Guangzhou, Guangdong 510182, China

[2]The Third Affiliated Hospital of Guangzhou Medical University, Guangzhou, Guangdong, China

[3]School of Health Sciences, Caritas Institute of Higher Education, Hong Kong SAR, China

[*] Corresponding author. Tel.: +86-020-81340659; Fax: +86-020-81340896.

E-mail addresses: zhouying0610@163.com

[†]Qing Luo and Chunqin Liu contributed equally to this work and share first authorship

1 Supplementary information of the WB-NRSs

Well-being Numerical Rating Scales (WB-NRSs)

Please indicate the degree of well-being you currently experience in each of the four areas indicated below. We also ask you to indicate the degree of overall well-being you perceive at this precise moment. You can answer using the following scale from 1 to 10 where 1 indicates a state of *absolute distress* and 10 a state of *complete well-being*.

Physical well-being

---

1 2 3 4 5 6 7 8 9 10

Psychological well-being

---

1 2 3 4 5 6 7 8 9 10

Relational well-being

---

1 2 3 4 5 6 7 8 9 10

Spiritual well-being

---

1 2 3 4 5 6 7 8 9 10

General well-being

---

1 2 3 4 5 6 7 8 9 10

幸福感数值评定量表

指导语：请说明目前您在以下四个方面感受到的幸福程度，同时指出您此刻感受到的总体幸福程度。请您用 1~10 的数值来回答，其中“1”表示绝对痛苦的状态，“10”表示完全幸福的状态。

生理幸福感

1 2 3 4 5 6 7 8 9 10

心理幸福感

1 2 3 4 5 6 7 8 9 10

社会幸福感

1 2 3 4 5 6 7 8 9 10

精神幸福感

1 2 3 4 5 6 7 8 9 10

总体幸福感

1 2 3 4 5 6 7 8 9 10
